# Chemical Characterization, Antioxidant Capacity and Anti-Oxidative Stress Potential of South American Fabaceae *Desmodium tortuosum*

**DOI:** 10.3390/nu15030746

**Published:** 2023-02-01

**Authors:** José-Luis Rodríguez, Paola Berrios, Zoyla-Mirella Clavo, Manuel Marin-Bravo, Luis Inostroza-Ruiz, Mariella Ramos-Gonzalez, Miguel Quispe-Solano, Maria S. Fernández-Alfonso, Olga Palomino, Luis Goya

**Affiliations:** 1 Faculty of Veterinary Medicine, Universidad Nacional Mayor de San Marcos, Lima 15021, Peru; 2 Faculty of Veterinary, Universidad Complutense de Madrid, 28040 Madrid, Spain; 3 Faculty of Biological Sciences, Universidad Nacional Mayor de San Marcos, Lima 15021, Peru; 4 Faculty of Pharmacy, Universidad Nacional Mayor de San Marcos, Lima 15021, Peru; 5 Faculty of Engineering in Food Industries, Universidad Nacional del Centro del Perú, Huancayo 12006, Peru; 6 Faculty of Pharmacy, Universidad Complutense de Madrid, 28040 Madrid, Spain; 7 Department of Metabolism and Nutrition, Spanish National Research Council (CSIC), Institute of Food Science, Technology and Nutrition (ICTAN), Jose Antonio Novais 10, 28040 Madrid, Spain

**Keywords:** plant antioxidants, phytochemicals, polyphenols, medicinal plants, vascular endothelium, neuroprotection

## Abstract

It has been proposed that oxidative stress is a pathogenic mechanism to induce cytotoxicity and to cause cardiovascular and neuronal diseases. At present, natural compounds such as plant extracts have been used to reduce the cytotoxic effects produced by agents that induce oxidative stress. Our study aimed to evaluate the antioxidant and cytoprotective capacity of *Desmodium tortuosum* (*D. tortuosum*) extract in the co- and pre-treatment in EA.hy926 and SH-SY5Y cell lines subjected to oxidative stress induced by tert-butylhydroperoxide (t-BOOH). Cell viability, reactive oxygen species (ROS), nitric oxide (NO), caspase 3/7 activity, reduced glutathione (GSH), glutathione peroxidase (GPx), glutathione reductase (GR), and molecular expression of oxidative stress biomarkers (SOD2, NRF2 and NFκB1) and cell death (APAF1, BAX, Caspase3) were all evaluated. It was observed that the *D. tortuosum* extract, in a dose-dependent manner, was able to reduce the oxidative and cytotoxicity effects induced by t-BOOH, even normalized to a dose of 200 µg/mL, which would be due to the high content of phenolic compounds mainly phenolic acids, flavonoids, carotenoids and other antioxidant compounds. Finally, these results are indicators that the extract of *D. tortuosum* could be a natural alternative against the cytotoxic exposure to stressful and cytotoxic chemical agents.

## 1. Introduction

The chemical products as xenobiotics can cause the production of ROS and other free radical that may result in inflammatory and fibrotic processes [1]. After absorption, the first tissue affected is the vascular system, especially the endothelia, this being the first step towards the development of vascular diseases [2]. Oxidative stress is capable of inducing vascular endothelial damage, which would produce a change in the vascular structure, this being one of the causes of diseases such as diabetes or nephropathy. Therefore, it is important to reduce this stressful effect on the vascular endothelium, with medicinal plants being a good therapeutic alternative [3,4,5]. During the last decade, studies have been published indicating that the antioxidant compounds of medicinal plants have the function of reducing endothelial damage and this would make them effective against cardiovascular diseases [6,7].

Neurodegenerative diseases, characterized by progressive dysfunction and cellular senescence of specific neuronal systems, involving structural and functional damage of neurons, may be caused by oxidative stress [1,8,9]. Recent mounting evidence suggests that oxidative stress in neuronal cells contributes to neuroinflammation, facilitated by the constant activation of microglia, hence inducing neuronal necrosis and apoptosis [8,9]. Since the onset of oxidative stress seems to be a main contributive cause of cardiovascular and neurodegenerative pathologies, protection of cells, tissues and organs against this challenging condition is the major goal of many studies dealing with nutritional and pharmacological prevention of pathologies.

During the last two decades, extended research on plant antioxidants, especially polyphenols, has unequivocally demonstrated their role as bioactive compounds that protect against oxidative stress and prevent or delay the onset of many pathologies [5,9,10]. For example, metabolic diseases, such as type 2 diabetes, or cardiovascular complications have endothelial damage as a common factor and, also, are characterized by an excess of free radical, oxidative stress and pro-inflammatory cytokines creating an oxidative and pro-inflammatory environment [11]. Some alternatives to counteract these effects are medicinal plants, such as *D. tortuosum,* which has antioxidant activity due to its high concentration in polyphenols, polyterpenes and flavonoids and their metabolites. In addition, continuing with the vascular endothelium, cocoa catechins are known to have a positive effect on healthy vascular function [10,12] which has been proclaimed since the pioneer studies from two decades ago [13,14,15], up to recent reviews [12,16,17]. However, the beneficial effect on cardiovascular function is not privative of flavanols or flavonoids in general [16] as we have already described before, also many other phenolic compounds or plant extracts have shown this potential [18,19]. Indeed, by using EA.hy926 cells, we have shown the protective effect of an extract from *Silybum marianum*, rich in flavonol derivatives known as flavonolignans, on cultured endothelial cells subjected to high glucose concentrations [20]. In the same model, *Vochysia rufa* stem bark extract, rich in reducing sugars and flavonoids, also showed significant protection against high glucose damage [21]. Similarly, in yerba mate and green coffee extracts, their main hydroxycinnamic acids and microbial metabolites prevented Tumor Necrosis Factor-alpha (TNF-α)-induced inflammation [22]. More recently, cocoa flavanols were proven to protect the same EA.hy926 cells against chemically induced oxidative stress [23]. All these studies confirm the protective effect on endothelial function of plant extracts and pure antioxidant compounds, as well as endorse the reliability of the cell culture model.

On the other hand, the preventive effects of polyphenolic antioxidants in aging and neurodegeneration associated with oxidative stress have been largely reported [1,9,24,25]. Since the culture of primary neurons is rather difficult, established cell lines have been widely used to test the neuro-regulatory effect of different bioactive compounds and their specific effects at the cellular and molecular levels. Neuroblastoma SH-SY5Y cells, derived from the SK-N-SH cell line, is one of the most commonly used neuronal-like cell cultures, and it has been recently validated as a simple reliable model of neuronal-like cells that is amenable to biological, biochemical and electrophysiological investigation [26]. In fact, using this human cell line as a neuronal cell culture model, the chemo-protective effect of an aqueous extract of cocoa phenolic compounds (mainly flavanols) against oxidative stress-induced neurodegeneration has been recently reported [27], as well as that of an extract from *Sambucus nigra* (elderflower), rich in flavonoids and hydroxycinnamic acids [28].

Thus, in this study, the polyphenolic chemical composition of the *D. tortuosum* extract was determined and to delineate the potential protective mechanisms through which *D. tortuosum* extracts protect endothelial and neuron cell function, two human cell lines, EA.hy926 and SH-SY5Y cells, were treated with t-BOOH a strong pro-oxidant used to induce oxidative stress in cell cultures.

## 2. Materials and Methods

### 2.1. Reagents

t-BOOH, glutathione reductase (GR), reduced (GSH) and oxidized (GSSG) glutathione, o-phthaldialdehyde, nicotine adenine dinucleotide phosphate reduced salt (NADPH), 2,4-dinitrophenylhydrazine, gentamicin, penicillin G and streptomycin, 2′,7′-dichlorofluorescin-diacetate (DCFH-DA), 4-amino-5-methylamino-2,7-difluorofluorescein-diacetate (DAF-FM-DA), 3-(4,5-dimethylthiazol-2-yl)-2,5-diphenyl-tetrazolium bromide (MTT), Dulbecco’s phosphate-buffered saline (DPBS, D8537) were obtained from Sigma-Aldrich (Madrid, Spain). Acetonitrile, methanol of high performance liquid chromatography (HPLC) grade, dimethyl sulfoxide (DMSO) of analytical grade and all other usual laboratory reagents were acquired from Panreac (Barcelona, Spain). Nucleo-spin-RNA, qPCRBIO-cDNA-synthesis, ICgreen-amplification-PCR, DMEM-culture-media and fetal bovine serum (FBS) were from Cultek (Madrid, Spain). The Apo-ONE^®^ Homogeneous Caspase-3/7 Assay kit was acquired from Promega (Madison, WI, USA). Bradford reagent was from BioRad Laboratories S.A (Hercules, CA, USA). All other chemical reagents used were of high-purity for cell and molecular biology and were available in the laboratory.

### 2.2. Plant Selection and Extract Preparation

The biological material was collected in the Monte Alegre district, Padre Abad province, Ucayali region, Peru coordinates 0498477LS-9030950LW, at an altitude of 194 m.a.s.l.; 10 kg of stems and leaves were collected (voucher number RS978). In the Ucayali Veterinary Institute-Pucallpa Regional Herbarium (HRUIP), the plants were dried, herbalized, and assembled, and the taxonomic verification of the species was carried out by comparison with existing samples and the use of a specialized bibliography, voucher number RS978. The obtained plants belonged to the species *D. tortuosum* of the Fabaceae family and were entered in the Herbarium under the registration number 12208.

The stems and leaves were collected and washed-and-dried in open air, then completely dried in an oven and reduced to a fine powder. The decoction was made with distilled water and the powdered plant material (10:1) in a beaker, heating until boiling and maintaining for twenty minutes. Plant material was filtered off and the aqueous extract was concentrated and lyophilized.

### 2.3. Chemical Characterization of Extract

Briefly, the sample (20 mg) was diluted with methanol (20 mL). The mixture was ultrasonicated for 10 min. Then, it was filtered through a 0.25 µm filter, and 3 µL were injected into a Dionex Ultimate 3000 (Thermo Scientific, Waltham, MA, USA) UHPLC system. Column was a Luna© Omega (Phenomenex Inc., Torrance, CA, USA) C18 100 Å, Phenomenex (150 × 2.1 mm, 1.6 µm), temperature 40 °C, flow rate 0.25 mL/min, with distilled water 1% formic acid and acetonitrile 1% formic acid eluents. The UHPLC system was coupled to a QExactive PLus mass spectrometer (Thermo Scientific, Waltham, MA, USA). Full Mass Spectrometry (MS) scan parameters were in the range of 120–1500 *m*/*z*, resolution 70,000, microscans 1, Automatic Gain Control (AGC) target of 1 × 10^6^, and maximum intensity (IT) of 100 ms. The parameters of the Resolution Type MS2 (MS2) resolution were 17,500, an AGC target of 2 × 10^5^, and a maximum IT of 50 ms. The ionization source parameters were Electrospray ionization (ESI) (negative/positive), spray voltage 2.5/3.0, temperature 280 °C, N_2_ (sheath gas flow rate: 40, aux gas flow rate: 10), gas heater temp of 350 °C, S-lens radio frequency (RF) level of 100, and a normalized collision energy of 20, 40, 60. The *m*/*z* values of the ions were detected in full ESI-MS (positive and/or negative) and the main fragments observed in the MS/MS spectra, the error in ppm is also indicated for the calculation of the molecular formula (≤5 ppm).

### 2.4. Cell Culture

Human EA.hy926 cells were a gift from Prof. Patricio Aller (CSIC, Madrid, Spain), and the SH-SY5Y cell line was a gift from Prof. Ignacio Torres Alemán (Instituto Cajal, Madrid, Spain), and later (same depositary batch) from Prof. Carlos Guillén, School of Pharmacy, University Complutense, Madrid, Spain. The cells were cultured and passaged in DMEM-F12 with FBS (10%) and 50 mg/L of gentamicin, penicillin and streptomycin. Cells were incubated in humid conditions with 5% CO_2_ and 95% air, at 37 °C; the culture medium was changed every other day.

Different concentrations of the *D. tortuosum* extract (1, 10, 25, 50, 100 and 200 μg/mL) dissolved in DMEM-F12 were added to microwell plates. To evaluate the protective effect of the *D. tortuosum* extract against oxidative stress, co- and pre-treatment were carried out. In the co-treatment assay, EA.hy926 and SH-SY5Y cells were simultaneously treated for 22 h with 100 µM t-BOOH plus the different concentrations of *D. tortuosum*; whereas in the pre-treatment assay cells were first treated with noted doses of extract for 18 h, then washed and submitted to a new media containing 200 µM t-BOOH for 4 h, after which the assay was performed [29].

### 2.5. Cell Viability Evaluation (MTT)

In this assay, it is observed if the mitochondria is active and is capable of reducing tetrazolium-MTT [30]. Briefly, after treatments, 0.5 mg/mL MTT as the final concentration in was added to each well for 2 h, during this time, metabolically active EA.hy926 and SH-SY5Y cells reduced the tetrazolium-MTT to a formazan-salt. Absorbance was measured at 540 nm (SPECTROstar BMG microplate reader (BMG Labtech, Ortenberg, Baden-Wurttemberg, Germany)). Cell viability is represented as % of control.

### 2.6. Intracellular ROS Production

Oxidative stress was assessed by the ROS intracellular production according to standardized protocols using the DCFH-DA-fluorescence assay [31]. DCFH-DA enters the cell and is hydrolyzed by esterases to allow the release of DCFH and its reaction with ROS to generate a fluorescent compound. Briefly, 10 µM of DCFH-DA was added to each well (2 × 10^5^ cells/well under incubation conditions) in a black multi-well plate for 30 min, and immediately measured in a fluorescent microplate reader (FLx800 Fluorimeter, BioTek, Winooski, VT, USA) at 485 nm/530 nm (λ excitation/λ emission).

### 2.7. Determination of Nitric Oxide (NO) Levels

NO levels were determined by direct measurement using the DAF-FM-DA assay. The cells were seeded in black 96-well plates at a rate of 8 × 10^4^ cells. After treatment, 1 mM DAF-FM-DA stock solution was added to each assay well to obtain a final concentration of 10 μM for 30 min. Then, the intensity of the fluorescent signal was measured in a microplate fluorescence reader (FLx800 Fluorimeter, BioTek, Winooski, VT, USA) at a λ excitation of 495 nm and λ emission of 515 nm [32].

### 2.8. Apoptotic Assay with Caspase 3/7 Activity

EA.hy926 and SH-SY5Y cells (15 × 10^3^ cells/well) were seeded in black 96-well plates. After treatment, Apo-ONE^®^ (Promega (Madison, WI, USA)) Caspase-3/7 was prepared and used according to the manufacturer’s instructions, for 60 min in the dark. Fluorescence (λ excitation/λ emission, 485/528 nm) was measured using a plate reader (FLx800, BioTek, Winooski, VT, USA). Data were evaluated as % of the control [32].

### 2.9. Antioxidant Defenses

#### 2.9.1. Reduced Glutathione (GSH)

GSH content was evaluated by a fluorometric assay [29]. The method takes advantage of the reaction of GSH with o-phthalaldehyde at pH 8.0. Fluorescence was measured at 340 nm/460 nm (λ excitation/λ emission). Fluorescence data were interpolated from a standard curve of pure GSH (5–1000 ng).

#### 2.9.2. Antioxidant Enzymes

Determination of GPx activity was based on the oxidation of GSH by GPx, using t-BOOH as a substrate, coupled to the disappearance rate of NADPH by GR [29]. GR activity was determined by following the decrease in absorbance due to the oxidation of NADPH utilized in the reduction in oxidized glutathione [29]. Protein concentration in the samples was measured by the Bradford reagent.

### 2.10. Molecular Assay by Real-Time PCR

After treatment, total RNA was obtained using the NucleoSpin^®^-RNA-Plus Kit (Macherey-Nagel, Germany) according to the manufacturer’s instructions. The total RNA was quantified using a Nano-Spectrophotometer (Microdigital, Seoul, Korea), obtaining A260/A280 ratios > 1.9–2.1 < in all the samples. cDNA synthesis was obtained from 1 μg of total RNA by retro-transcription using the qPCRBIO cDNA Synthesis Kit (PCRBiosystems, Wayne, PA, USA). Finally, the cDNA was diluted in nuclease-free water (*v*:*v*, 1:10) and stored at −80 °C. Real-time PCR (qPCR) assays for SOD2, NRF2, NFκB1 (genes from oxidative stress-antioxidant), APAF1, BAX, and Caspase 3 (genes from cell death) were performed using a real-time PCR system (BioRad CFX, Hercules, CA, USA), using the ICgreen Mastermix (Nippon Genetics, Duren, Germany) according to the manufacturer’s instructions. For qPCR, it was necessary to use primers with concentrations of 400 nM, and the thermocycling protocol was: 95 °C for 2 min, 40 cycles of 5 s at 95 °C and 30 s at 60 °C.

Forward and reverse primers were SOD2: ‘CCACTGCTGGGGATTGATGT’ ‘CGTGGTTTACTTTTTGCAAGCC’; NRF2: ‘CTGGTCATCGGAAAACCCCA’ ‘TCTGCAATTCTGAGCAGCCA’; NFκB1: ‘TTTTCGACTACGCGGTGACA’ ‘GTTACCCAAGCGGTCCAGAA’; APAF1: ‘TCTTCCAGTGGTAAAGATTCAGTT’ ‘CGGAGACGGTCTTTAGCCTC’; BAX: ‘CCCCCGAGAGGTCTTTTTCC’ ‘CCTTGAGCACCAGTTTGCTG’; Caspase3: ‘GTGGAGGCCGACTTCTTGTA’ ’TTTCAGCATGGCACAAAGCG’. GAPDH was used as a housekeeping gene and extracting the efficiencies from the raw data using the LinRegPCR software [33].

### 2.11. Statistics

Analysis of the data obtained from the cell culture studies was performed with one-way ANOVA followed by Tukey’s post hoc test, and the level of significance was *p* < 0.05 using the GraphPad Prism version 7.0 program (Boston, MA, USA).

## 3. Results

### 3.1. Chemical Analysis of D. Tortuosom Extract

Thirty compounds were identified in *D. tortuosum* based on their relative retention time, mass spectra and commercial standards. Table 1 shows the retention time (RT), molecular formula, accurate mass of the molecular ion (M – H)^−^ after negative–positive ionization, and MS^2^ fragments of the main compounds identified in *D. tortuosum* by Ultra High Perfomance Liquid Chromatography (UHPLC) UHPLC/MS (Supplementary Figure S1). The largest number of compounds identified were phenolic acids and flavonoids, and to a lesser extent compounds derived from linoleic acid, and others.

### 3.2. Cell Viability

**Figure 1:**
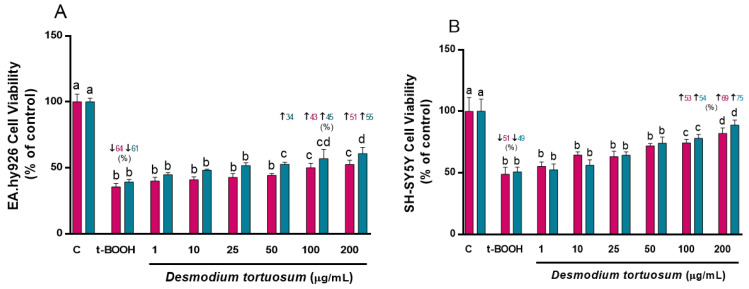
Cytoprotective effects by *D. tortuosum* extract on EA.hy926 (**A**) and SH-SY5Y (**B**) cells after co-treatment (red bars, █) and pre-treatment (blue bars, █) periods. Data are presented as % control, and as mean ± SEM of six independent experiments. ^a,b,c,d^ Different letters show significance between groups at *p* < 0.05. ↓ represents percentage decrease with respect to control, ↑ represents percentage increase with respect to t-BOOH.

The first parameter to ascertain the potential cyto-protection of the *D. tortuosum* extracts was cell viability after oxidative stress. *D. tortuosum* extracts at 1, 10, 25, 50, 100 and 200 µg/mL were tested for their cyto-protective capacity. As depicted in Figure 1, exposure to t-BOOH produced a significant decrease in cell viability of around 62% in EA.hy926 cells and 50% in SH-SY5Y cells. Increasing the concentrations of extract evoked a partial but significant dose-dependent recovery of cell viability in the two cell lines for both co- and pre-treatment. *D. tortuosum* extracts at 100 and 200 µg/mL significantly increased SH-SY5Y cell viability for both co- and pre-treatment (Figure 1B), and in the pre-treatment of EA.hy926, whereas concentrations above 50 µg/mL were necessary to significantly recover EA.hy926 cell viability with co-treatment (Figure 1A). The highest recovery of cell viability from the oxidative stress was observed at co- and pre-treatment with 200 µg/mL extract in SH-SY5Y cells (Figure 1B). Once the cyto-protective effect of some of the tested concentrations of the extract was ensured, the study of the redox status and antioxidant response was carried out.

### 3.3. Intracellular ROS Production

**Figure 2:**
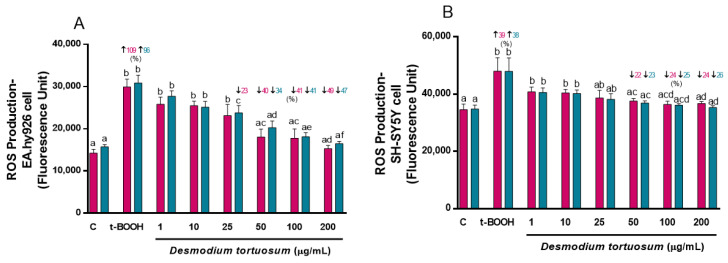
Antioxidant effects by *D. tortuosum* extract on ROS production induced by t-BOOH in EA.hy926 (**A**) and SH-SY5Y (**B**) cells after co-treatment (red bars, █) and pre-treatment (blue bars, █) periods. ROS production was measured as fluorescence units. Data are presented as mean ± SEM of six independent experiments. ^a,b,c,d,e,f^ Different letters show significance between groups at *p* < 0.05. ↑ represents percentage increase with respect to control, ↓ represents percentage decrease with respect to t-BOOH.

The addition of t-BOOH to cell cultures induced a remarkable increase in ROS generation of around 100% in EA.hy926 and around 40% in SH-SY5Y cells, very similar for both types of treatments, ensuring the reliability of the model for oxidative damage (Figure 2). The same extract concentrations (1–200 µg/mL) tested for cell viability were also assayed for their ROS-quenching capacity. Similar to the assay of cell viability, a significant dose-dependent reduction in ROS production was observed with increasing doses of the extract and, in the case of EA.hy926 cells, a decline in ROS almost to control pre-stress values was reached with the highest tested concentration of 200 µg/mL of *D. tortuosum* extract (Figure 2A). The two highest extract concentrations, 100 and 200 µg/mL, were also efficient in preventing ROS overproduction induced by t-BOOH in SH-SY5Y cells. The results clearly indicate that both co- and pre-treatment with extracts from *D. tortuosum* in the range of 50–200 µg/mL, significantly reduced ROS production induced by oxidative stress in these two cell lines (Figure 2).

Since the highest protection against an oxidative challenge for both co- and pre-treatment approaches was obtained with the three highest concentrations of the extract, 50, 100 and 200 µg/mL, these three doses were tested for the rest of the oxidative stress biomarkers.

### 3.4. Determination NO Levels

**Figure 3:**
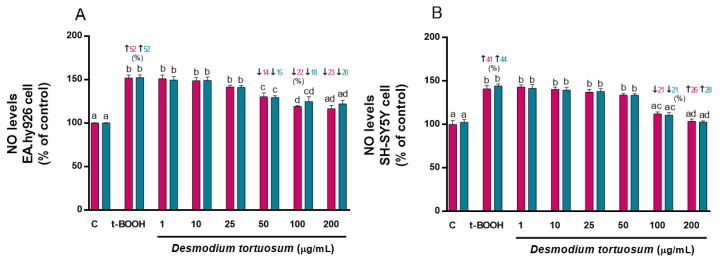
*D. tortuosum* extract effect on NO levels induced by t-BOOH in EA.hy926 (**A**) and SH-SY5Y (**B**) cells after co-treatment (red bars, █) and pre-treatment (blue bars, █) periods. NO levels are presented as % of control-change. Data represent the mean ± SEM of six independent experiments. ^a,b,c,d^ Different letters show significance between groups at *p* < 0.05. ↑ represents percentage increase with respect to control, ↓ represents percentage decrease with respect to t-BOOH.

When t-BOOH was added to cell cultures, an increase in NO levels was observed in EA.hy926 (around 50%) and SH-SY5Y cells (around 40%) (Figure 3), while the extracts of *D. tortuosum* in the highest doses prevented the effect of t-BOOH. In EA.hy296 cells, doses of 50, 100, and 200 µg/mL of *D. toruosum* reduced NO levels that were previously induced by t-BOOH; and in SH-SY5Y cells this effect could only be observed with the 100 and 200 µg/mL doses of *D. tortuosum* (Figure 3).

### 3.5. Apoptotic Assay: Caspase 3/7 Activity

**Figure 4:**
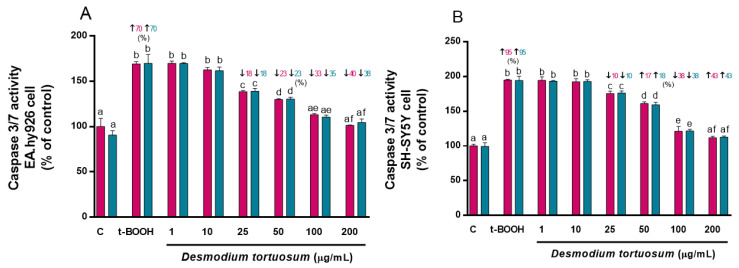
*D. tortuosum* extract effect on Caspase 3/7 activity induced by t-BOOH in EA.hy926 (**A**) and SH-SY5Y (**B**) cells after co-treatment (red bars, █) and pre-treatment (blue bars, █). Caspase 3/7 activity normalized as % of control-change, and the data are presented as mean ± SEM of six independent experiments. ^a,b,c,d,e,f^ Different letters show significance between groups at *p* < 0.05. ↑ represents percentage increase with respect to control, ↓ represents percentage decrease with respect to t-BOOH.

The capacity of *D. tortuosum* extract to reduce the apoptotic effects produced by t-BOOH was evaluated. The activity of the caspase 3/7 enzyme was increased by the effect of t-BOOH in EA.hy926 cells by 70% (co- and pre-treatment), and in SH-SY5Y cells by 95% (co- and pre-treatment) (Figure 4). *D. tortuosum* was able to reduce the apoptotic activity induced by t-BOOH in both cell lines from doses of 25, 50, 100, to 200 µg/mL; this effect was similar for both co- and pre-treatment with *D. tortuosum* (Figure 4).

### 3.6. Antioxidant Defenses

#### 3.6.1. GSH Concentration

**Figure 5:**
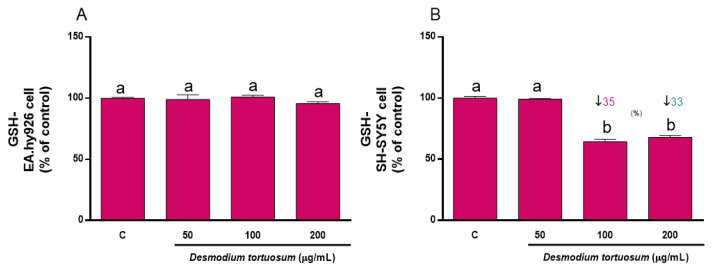
Direct effect of *D. tortuosum* extract on GSH levels in EA.hy926 (**A**) and SH-SY5Y (**B**) cells after a 22 h of treatment period. GSH levels was determined as % of control-change, and represent the mean ± SEM of four independent experiments. ^a,b,c^ Different letters show significance between groups at *p* < 0.05. ↓ represents percentage decrease with respect to control.

The concentration of GSH was determined as a reliable biomarker of the intracellular non-enzymatic antioxidant defenses. Since an acute treatment with pure compounds or extracts rich in natural antioxidants might evoke changes in the steady-state level of GSH that might affect its response to induced oxidative stress, both cell lines were subjected to a direct treatment for 22 h with the three extracts. Figure 5 shows that 50–200 µg/mL of extract did not induce any change in the basal concentration of GSH of EA.hy926 cells, whereas 100–200 µg/mL evoked a significant decrease in SH-SY5Y cells, indicating that neuronal-like cells were more sensitive to the presence of the extract concentrations for 22 h.

**Figure 6:**
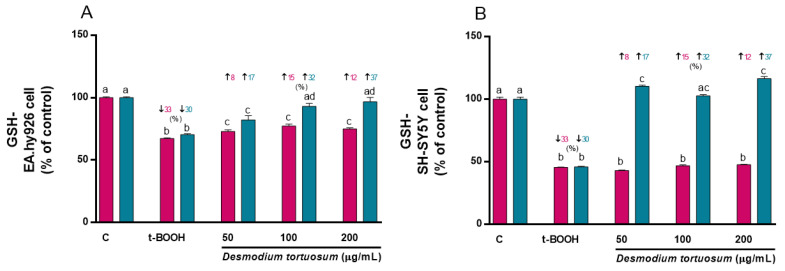
Effect of *D. tortuosum* extract on GSH levels altered by t-BOOH in EA.hy926 (**A**) and SH-SY5Y (**B**) cells after co-treatment (red bars, █) and pre-treatment (blue bars, █). GSH levels were determined as % of control-change, and represent the mean ± SEM of four independent experiments. ^a,b,c,d^ Different letters show significance between groups at *p* < 0.05. ↓ represents percentage decrease with respect to control, ↑ represents percentage increase with respect to t-BOOH.

Treatment of EA.hy926 and SH-SY5Y cells with 100 µM t-BOOH for 22 h (co-treatment) or with 200 µM t-BOOH for 4 h (pre-treatment) significantly reduced the cell GSH concentration (Figure 6A,B). There was a slight but significant increase in GSH in EA.hy926 cells subjected to co-treatment with the extracts; however, a significant dose-dependent recovery was observed when endothelial cells were pre-treated with the extract concentrations prior to exposure to the potent pro-oxidant (Figure 6A, pre-treatment). In SH-SY5Y cells, none of the three tested doses of extract recovered the depleted GSH when they were added simultaneously (co-treatment) to the pro-oxidant (Figure 6B). Nevertheless, when neuroblastoma cells were pre-treated with the extract prior to the stress, all three concentrations were capable of fully preventing the GSH decrease (Figure 6B), indicating a chemo-preventive effect of *D. tortuosum* on neuronal-like damage.

The results indicate that a pre-treatment with the extracts of *D. tortuosum* was effective in reducing cell death and quenching ROS and NO over-production, as well as diminishing caspase 3/7 activity, and significantly preventing the intense depletion of GSH induced by oxidative stress in both EA.hy926 and SH-SY5Y cells.

#### 3.6.2. Antioxidant Enzymes

**Figure 7:**
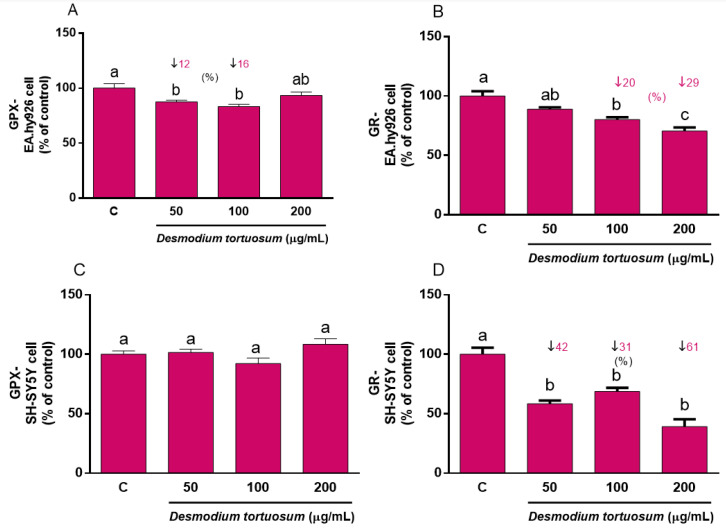
Direct effect of *D. tortuosum* extract on GPX and GR activities in EA.hy926 (**A**,**B**) and SH-SY5Y (**C**,**D**) cells after a 22 h of treatment period. GPX and GR activities were determined as % of control-change, and represent the mean ± SEM of four independent experiments. ^a,b,c^ Different letters show significance between groups at *p* < 0.05. ↓ represents percentage decrease with respect to control.

As the main antioxidant enzymes, evaluation of GPx and GR activity guarantees an archetypal response of the antioxidant system to a stressful challenge. As in the case of GSH, acute treatment with pure phytochemicals or natural extracts rich in antioxidants might evoke changes in the basal pre-stress activity of GPx and GR that might affect its further response to induced oxidative stress; thus, both cell lines were firstly subjected to a direct treatment for 22 h with the three extract doses. A slight but significant decrease in GPx activity was observed after treatment with 50–100 µg/mL extract in EA.hy926 cells (Figure 7A); similarly, a reduced activity of GR was found when EA.hy926 cells were treated with 100–200 µg/mL (Figure 7B). Direct treatment of SH-SY5Y cells with *D. tortuosum* extract for 22 h did not evoke any change in GPx activity (Figure 7C), whereas all three tested doses (50–200 µg/mL) induced a significant decrease in GR activity (Figure 7D), similar to what was observed in endothelial cells.

**Figure 8:**
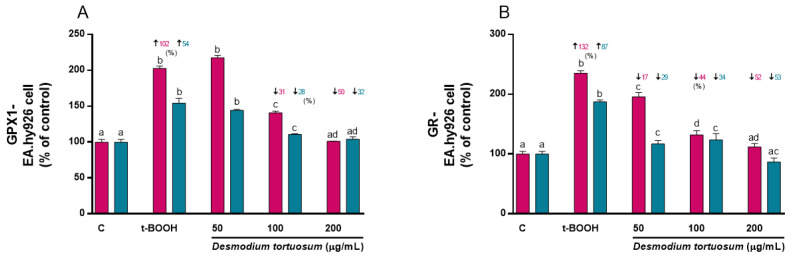
Effect of *D. tortuosum* extract on GPx (**A**) and GR (**B**) activity altered by t-BOOH in EA.hy926 cells after co-treatment (red bars, █) and pre-treatment (blue bars, █) periods. Data represent the mean ± SEM of three independent experiments. ^a,b,c,d^ Different letters show significance between groups at *p* < 0.05. **↑** represents percentage increase with respect to control, ↓ represents percentage decrease with respect to t-BOOH.

Treatment of EA.hy926 cells with 100 µM t-BOOH for 22 h evoked a 100% increase in GPx activity (Figure 8A), whereas treatment with 200 µM t-BOOH for 4 h provoked a 50% enhancement of the enzyme’s activity (Figure 8A). This result confirms the expected response of GPx to face the over-production of ROS induced by t-BOOH in EA.hy926 cells. In agreement with the GPx increase, GR activity was also stimulated by 100 µM of the pro-oxidant for 22 h to more than 100% (Figure 8B), and around two-fold by 200 µM of t-BOOH for 4 h (Figure 8B). This result of GR ensures appropriate recycling of GSSG to GSH for re-utilization. Remarkably, both co- and pre-treatment of EA.hy926 cells with 100–200 µg/mL of the extract evoked a significant reduction in the enhanced GPx activity, that was dose-dependent in the co-treatment, and reverted to basal activity at the end of the stress period (Figure 8A). Similarly, a dose-dependent rescue of the altered GR activity was observed when EA.hy926 cells were co- or pre-treated with the three extract concentrations (Figure 8B).

**Figure 9:**
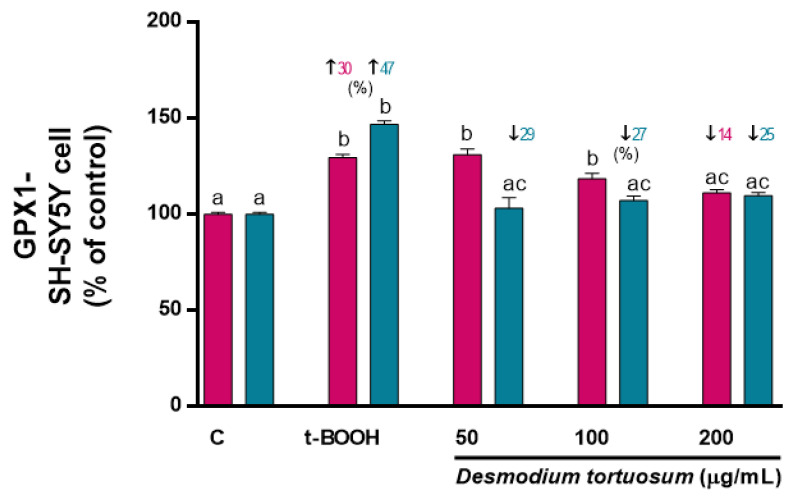
Effect of *D. tortuosum* extract on GPx altered by t-BOOH on SH-SY5Y cells after co-treatment (red bars, █) and pre-treatment (blue bars, █) periods. Data represent the mean ± SEM of three independent experiments. ^a,b,c^ Different letters show significance between groups at *p* < 0.05. ↑ represents percentage increase with respect to control, ↓ represents percentage decrease with respect to t-BOOH.

Treatment of SH-SY5Y cells with 100 µM t-BOOH for 22 h or with 200 µM t-BOOH for 4 h provoked a significant enhancement of GPx and GR activity (Figure 9), confirming the predictable response of both enzymes to face the over-production of ROS induced by t-BOOH and the suitable reprocessing of GSSG to GSH for re-utilization in SH-SY5Y cells. Co-treatment with the extract did not evoke a significant rescue of the enhanced GPx activity, whereas pre-treatment of the SH-SY5Y cells with the three doses of *D. tortuosum* extract remarkably reverted the stimulated GPx activity to the control pre-stress values (Figure 9). Unexpectedly, no significant changes in GR activity were found in the SH-SY5Y cells treated with 100 µM t-BOOH (co-treatment) or 200 µM t-BOOH (pre-treatment). As GR activity was very low in all conditions, the assay was not sensitive enough to detect any measurable changes in enzyme activity (data not shown). Overall, EA.hy926 cells were more robust and responsive to stressful conditions than SH-SY5Y cells but, in general, both co- and pre-treatment of endothelial and neuronal-like cells with the *D. tortuosum* extract significantly prevented the permanent enhancement of both antioxidant enzyme activities, especially GPx (Figure 8 and Figure 9).

### 3.7. Gene Expression of Oxidative–Antioxidative and Cell Death Biomarkers

Genes related to oxidative–antioxidative (SOD2, NRF2 and NFκB1) and cell death (APAF1, BAX and Caspase3) proteins were evaluated for the effect of t-BOOH or *D. tortuosum* extract (co- and pre-treatment) on EA.hy926 and SH -SY5Y cells.

**Figure 10:**
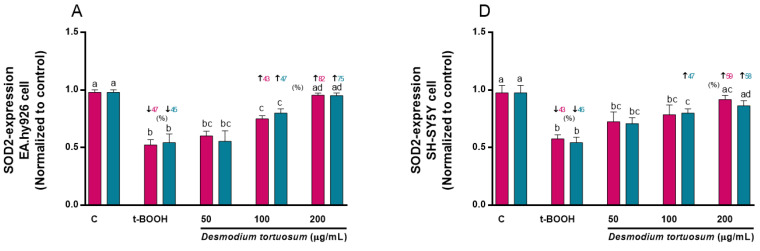

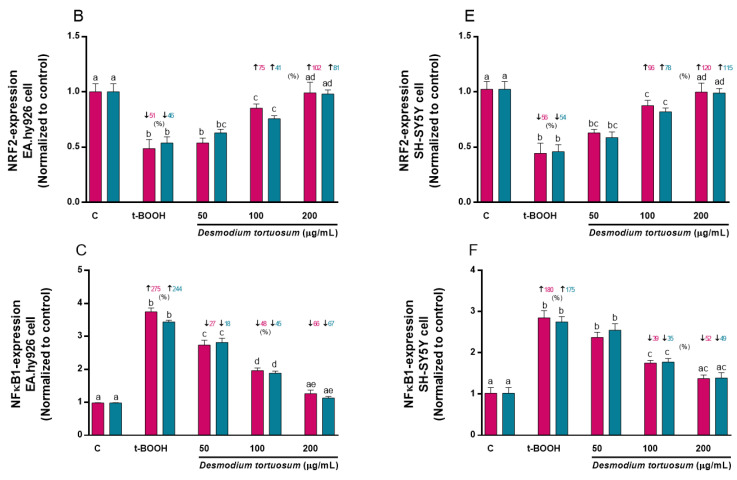
Effect of *D. tortuosum* extract (50, 100, and 200 µM) on the expression of oxidative-antioxidative genes (SOD2, NRF2, NFκB1) in EA.hy926 (**A**–**C**) and SH-SY5Y (**D**–**F**) cells after 24 h co-treatment (red bars, █) and pre-treatment (blue bars, █) periods. The gene expression was determined as control-normalized value, and represented as the mean ± SEM of four independent experiments. ^a,b,c,d^ Different letters show significance between groups at *p* < 0.05. **↓** represents percentage decrease or **↑** represents percentage increase with respect to control or with respect to t-BOOH.

Molecular expression of SOD2 was decreased by around 45% due to the effect of t-BOOH in both cell types, but this effect was reversed by the effect of the highest concentration of the *D. tortuosum* extract (200 µg/mL) in EA.hy926 and in SH-SY5Y cells (Figure 10A,D). Likewise, NRF2 gene levels decreased by around 50% and 55% due to the effect of t-BOOH in EA.hy926 and SH-SY5Y cells, respectively; while the 100 and 200 µg/mL doses, in both cell types, had the ability to significantly reduce the effect of t-BOOH (Figure 10B,E). Moreover, t-BOOH was able to increase NFκB1 expression levels above 250% and 175% in EA.hy926 and SH-SY5Y cells, respectively; and the *D. tortuosum* extract at all its doses reduced this effect in EA.hy926 cells, while only at 100 and 200 µg/mL of *D. tortuosum* in SH-SY5Y cells (Figure 10C,F).

**Figure 11:**
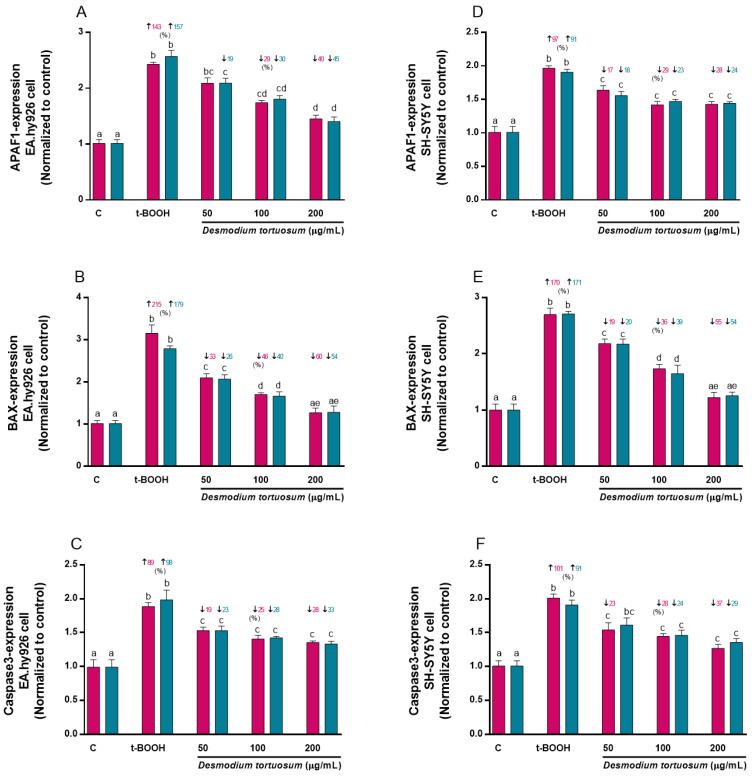
Effect of *D. tortuosum* extract (50, 100, and 200 µM) on the expression of cell death genes (APAF-1, BAX, Caspase3) in EA.hy926 (**A**–**C**) and SH-SY5Y (**D**–**F**) cells after 24 h co-treatment (red bars, █) and pre-treatment (blue bars, █) periods. The gene expression was determined as control-normalized values, and is represented the mean ± SEM of four independent experiments. ^a,b,c,d,e^ Different letters show significance between groups at *p* < 0.05. ↓ represents percentage decrease or ↑ represent percentage increase with respect to control or with respect to t-BOOH.

The molecular expression of APAF1 was significantly increased above 140% and 90% by the effect of t-BOOH in EA.hy926 and SH-SY5Y cells, respectively; but this effect was reversed by the effect of the concentrations of 50 (only pre-treatment in EA.hy926 cells), 100 and 200 µg/mL of *D. tortuosum* in EA.hy926 and SH-SY5Y cells, (Figure 11A,D). Likewise, BAX mRNA expression was significantly increased by the effect of t-BOOH by 215% (co-treatment) and 179% (pre-treatment) in the EA.hy926 cells, and around 170% in the SH-SY5Y cells; while all doses of *D. tortuosum*, in both cell types had the ability to significantly and dose-dependently reduce the effect of t-BOOH (Figure 11B,E). It was also observed that t-BOOH induced Caspase3 expression above 89% in both cell types, which was reduced by the effect of the *D. tortuosum* extract (50, 100 and 200 µg/mL) in both cell types (Figure 11C,F).

## 4. Discussion

In this study, an aqueous extract of *D. tortuosum* was prepared and its main phenolic compounds were characterized by UHPLC/MS, showing phenolic acids, flavonoids such as flavones, flavanones and flavanols, carotenoids and others antioxidant compounds (Table 1) as the main compounds with bioactive potential. The extract showed significant antioxidant capacity in vitro and effects in endothelial and neuronal-like cell culture that include the regulation on ROS production and NO concentration, caspase 3/7 activity and a remarkable anti-oxidative stress protection and molecular regulation of biomarkers of oxidative stress and cell death. All these effects support the use of the plant since ancient times in traditional medicine.

The range of doses of the D. *tortuosum* extract to test the anti-oxidative stress potential was selected according to previous data from other studies working with plant extracts, foodstuff and juices. A concentration of 35 µM of flavanol epicatechin was found in rat serum 1 h after oral administration of 172 µmol epicatechin per Kg of body weight [34]. Similarly, levels of 30–40 µM of cranberry phytochemicals have been detected in plasma after the intake of cranberry juice [35,36]. Hence, the range of *D. tortuosum* extract concentrations tested is not far from realistic; in fact, in previous works we have report the protective activity of *Vochysia rufa* (0.5–100 µg/mL) [21], *Silybum marianum* (5–25 µg/mL) [20], and cocoa extract (2.5–20 µg/mL) [23] in EA.hy926 cells. Additionally, we have recently reported that doses of 5–25 µg/mL of a *Sambucus nigra* extract [28] and 25–200 µg/mL of an aqueous extract of cocoa phenolic compounds [27] protect SH-SY5Y cells from oxidative stress.

Previous results have indicated that the treatment of EA.hy926 cells with t-BOOH is an excellent oxidative model in cell culture [20,23,37]. Similarly, very recently we have also established a oxidative model in SH-SY5Y cells by a comparable treatment with the same pro-oxidant, t-BOOH [27,28]. As most organic peroxides, t-BOOH decompose to other alkoxyl and peroxyl radicals in a reaction assisted by metal ions that can generate ROS, including H_2_O_2_ [29]. If the over-production of ROS is long-lasting, damage to macromolecules, proteins, lipids and DNA, might be excessive and irreversibly endanger cell viability, as observed in t-BOOH-treated cells. However, under these stressful conditions, significant inhibition of t-BOOH-induced cytotoxicity when both EA.hy926 and SH-SY5Y cells were pre- or co-treated with plant extracts at realistic doses for 20 h indicated that the integrity of the stressed cells was remarkably protected against the potent oxidative challenge. The relevant amount of the bioactive phenolics in the extract was effective enough for partial but significant dose-dependent cell protection, although the different responses to co- and pre-treatment suggest a differential sensitivity of the two cell types to the extracts in stressful conditions; pre-treatment being more effective in both cell lines. As reported above, a similar cytoprotective capacity has been reported with other phenolic extracts in both cell lines, EA.hy926 [20,21,23] and SH-SY5Y cells [27,28].

Assessment of ROS generation is a reliable index of the redox status as well as the oxidative damage to living cells [29]. The addition of t-BOOH to either cell line in both, co- and pre-treatment conditions, evoked a significant increase in ROS generation that might be the main cause for the increased cell death. The significant dose-dependent reduction in ROS induced by t-BOOH observed with co- and pre-treatment with extract in both cell lines unequivocally support the antioxidant nature of the phenolic components and could be a primary explanation for the reduced oxidative stress and subsequent cell protection. Interestingly, a comparable ROS-quenching capacity has been reported not only in both EA.hy926 and SH-SY5Y cells as referred above [20,21,23,27,28], but also in cultured hepatic cells [38,39,40,41,42], clearly indicating that this chemo-protective effect is not specific of a particular cell type or tissue but an systemic anti-oxidative stress capacity of natural antioxidants.

After administering t-BOOH to both cell cultures, NO levels increased significantly. This similar effect of increasing NO in cell cultures by cytotoxic substances has been previously reported [32,43]. The NO concentrations were reduced in a dose-dependent manner by the effect of *D. tortuosum* from 50 µg/mL in both co- and pre-treatment in EA.hy926 cells, and from 100 µg/mL in co- and pre-treatment in SH-SY5Y cells. NO is a signaling molecule that plays an important role in prolonging inflammation and immune responses. *D. tortuosum* and other extracts could act as NO scavengers or inhibitors of its production, through the inhibition of NO activity, inducible nitric oxide synthase (iNO) or through free radical scavenging activities [44,45].

Many forms of cellular stress can lead to cell death, through intracellular stress or mitochondrial dysfunction [46]. In this study it was observed that t-BOOH is capable of inducing oxidative stress by increasing the levels of ROS and NO. This effect may have produced an increase in the caspase 3/7 activity, enzymes involved in cell death and evaluated in this study. It is also known that under conditions of oxidative stress, high levels of ROS (superoxide, hydroxyl radical and hydrogen peroxide) are generated, which induce cell damage and cell death [47]. This cell death often involves the induction of apoptosis through the activation of caspase enzymes [48]. In this study, the high caspase 3/7 activity found in EA.hy926 and SH-SY5Y cells was reduced in a dose-dependent manner by the effect of the *D. tortuosum* extract starting at 25 µg/mL for both pre- and co-treatment. Similar effects have been reported in other studies, where they observed that natural compounds with a high phenolic content reduced the activity of caspase enzymes in EA.hy925 [49,50] and SH-SY5Y cells [51,52].

The best biomarker of the cell redox status is the concentration of GSH; thus, GSH depletion or reduction indicates increased intracellular oxidation and precarious redox status, whereas a balanced GSH concentration positions the cell in an advantageous situation to face potential oxidative stress [53]. Concentration of GSH is tightly regulated within the cell and direct exposure to plant extracts at non-toxic concentrations does not usually evoke significant changes in basal GSH levels [20,21,22,29]. Thus, the decline in GSH concentrations observed in SH-SY5Y cells treated with 100–200 µg/mL extract may be a consequence from the direct conjugation of some extract compounds to GSH, a fact previously reported for flavonoids, such as catechin [54] and epigallocatechin-3-gallate [55]. This direct conjugation might be only relevant in the case of direct treatment with the highest concentrations tested because of the larger amount of flavonoids and other antioxidants in the extract that are not consumed to face the oxidative stress. On the other hand, the decreased GSH concentration induced by t-BOOH suggests a state of oxidative stress that might result in irreparable oxidative damage to macromolecules: lipids, proteins and nucleic acids. This hazardous situation was dose-dependently prevented by the pre-treatment in EA.hy926 cells and completely prevented by pre-treatment with all three doses in the SH-SY5Y cells. The results suggest that the condition of co-treatment, with the continuous presence of a strong pro-oxidant during the whole assay, was too severe for the extract compounds to recover the consumed GSH to suitable levels in the cells. In any case, the response in the pre-treatment assay is in agreement with reports of other plant extracts rich in phenolic antioxidants such as *Silybum marianum* [20], *Vochysia rufa* [21] and green coffee [22] in EA.hy926 cells, as well as cocoa [27] and *Sambucus nigra* [28] in SH-SY5Y cells. This outcome is essential since preserving GSH concentrations above an appropriate threshold while struggling against a stressful situation represents a crucial advantage for cell survival.

Activities of GPx and GR enzymes are essential to balance the cellular redox state. GPx induces the reduction in cell-damaging peroxide species, along with the conversion of GSH to oxidized glutathione [29,53], whereas GR recycles oxidized glutathione back to GSH [29,53], recovering the steady state of cellular GSH. The increase in GPx and GR activities observed after the noted treatments with t-BOOH unambiguously indicates a positive response of the cell’s defense system to face oxidative stress [29,38,39,40,41,42]. Consequently, during or after induced oxidative stress the antioxidant defense system of the cells pre-treated with *D. tortuosum* extract rapidly returned to a steady-state condition minimizing cell damage and, thus allowing the cell to deal with further oxidative insults in conditions that are more favorable. We have previously demonstrated a similar chemo-protective response of antioxidant defense enzymes by other antioxidant extracts in the same two cell lines [20,21,23,27,28].

Oxidative stress is one mechanisms through which cells respond by activating cell survival or cell death pathways. Initially, cell tries to respond to oxidative damage in a positive way, but if the damage too extensive the cell death mechanism will be activated. In other words, the alteration of the oxidant–antioxidant mechanisms will activate cell survival or death mechanisms in the cells [56]. In this study, we observed that the molecular expression of the antioxidant biomarkers SOD2 and NRF2, and the oxidant biomarker NFκB1 are altered by the effect of t-BOOH and are completely restored with a 200 µg/mL dose of *D. tortuosum*. Likewise, it was observed that molecular biomarkers of cell death, such as APAF1, BAX and Caspase3, were overexpressed by the effect of t-BOOH, and this effect was reversed by the various concentrations of *D. tortuosum* in the EA.hy926 and SH-SY5Y cells. Therefore, we can conclude that the *D. tortuosum* extract has an antioxidant cytoprotective effect and a direct or indirect antiapoptotic effect (through antioxidant mechanisms). In other studies where natural extracts have been used, this antioxidant–antiapoptotic association effect has also been observed. This is the case of the increase in SOD2 levels and the decrease in the expression of BAX and Caspase3 proteins due to the effect of the *Scrophularia buergeriana* extract in the SH-SY5Y cells [57]. Furthermore, the genus *Astragalus* was reported to increase SOD levels and decrease NFκB activity in EA.hy926 cells [58]. Likewise, in cell cultures, the ability of natural extracts to increase the levels of SOD and NRF2, two molecules involved in cell antioxidant activity, has been observed [59,60].

In general, the response of EA.hy926 cells to stress was more constant and robust than that of the SH-SY5Y cells and the protective effect of the *D. tortuosum* extract was more efficient as a pre-treatment versus co-treatment. Overall data indicate that, under chemically induced oxidative stress, treatment of endothelial and neuronal-like cells with the *D. tortuosum* extract rich in antioxidant compounds reduces ROS production, NO generation, caspase 3/7 activity, and limits GSH depletion resulting in a restricted requirement for antioxidant enzyme activity. Likewise, it was observed that there is an important association between the expression of antioxidant molecules and the decrease in molecules that induce cell death. This inclusive biochemical and molecular response evoked by the bioactive extract could systematically explain the observed endothelial and neuronal-like cyto-protection.

## 5. Conclusions

The extract of *D. tortuosum* is rich in phenolic compounds with antioxidant capacity. This work demonstrates that the doses (50, 100 and 200 µg/mL) of the extract contributed to the cytoprotection of EA.hy926 endothelial and SH-SY5Y neuronal cells subjected to oxidative damage by t-BOOH, through the regulation of ROS, NO, GSH, antioxidant enzyme activity, caspase3/7 activity, and molecular biomarkers from oxidative stress and cell death. Taking into account all the data, it can be concluded that the treatment of EA.hy926 and SH-SY5Y cells with the *D. tortuosum* extract (from 50 µg/mL) practically normalizes (200 µg/mL) the antioxidant defense system of the cells after oxidative stress. More studies are needed to evaluate the mechanism of action and biological activity in vivo of *D. tortuosum*, before categorically concluding the potential protective effect of this botanic extract in animals and humans.

## Figures and Tables

**Table 1:** Identification of bioactive compounds detected by UHPLC/MS in the *D. tortuosum* extract.

Identified Compound	Retention Time (min)	Molecular Formula	MS-ESI−	MS2	MS-ESI+	MS2	Nominal Mass
**HYDROXYCINNAMIC ACIDS AND HYDROXYCINNATES**							
4-Coumaric acid	8.79	C_9_H_8_O_3_	163	119, 93			164
**CAROTENOIDS**							
Loliolide	9.60	C_11_H_16_O_3_			197	179, 161,135, 133	196
**FLAVONES**							
6-C-xylosyl-8-C-galactosylapigenin	8.16	C_26_H_28_O_14_	563	473,443,383	565	547,529,511	564
Vitexin-2″-O-rhamnoside	8.66	C_27_H_32_O_15_	577	457,413,341,323	579	433, 415, 367, 337	578
Vitexin	9.55	C_21_H_20_O_10_	431	341, 311, 283, 268			432
Isovitexin	8.85	C_21_H_20_O_10_	431	341,323, 311, 295	433	415, 397, 379, 367	432
Saponarin	8.10	C_27_H_30_O_15_	593	473, 431, 311, 297	595	433,415, 367, 337	594
luteolin-7-glucoside	9.03	C_21_H_20_O_11_	447	357,285,256			448
Luteolin-6-C-glucoside	8.33	C_21_H_20_O_11_	447	429, 357, 327	449	431, 383, 353, 329	448
6-C-arabinosyl-8-C-β-D-xylosylapigenin	8.66	C_25_H_26_O_13_	533	443, 413, 383, 353	535	517, 499, 481, 469	534
**FLAVANONES**							
Naringenin	11.62	C_15_H_12_O_5_	271	177, 151, 119			272
Prunin	9.53	C_21_H_22_O_10_	433	271, 177, 151			434
8-Prenylnaringenin	13.79	C_20_H_20_O_5_	339	245, 233, 219	341	285,183,165	340
2’,4’,5,7-Tetrahydroxy-8-prenylflavanone	13.32	C_20_H_20_O_6_	355	193,161,149	357	301, 283	356
**FLAVONOLS**							
Hyperoside	8.95	C_21_H_20_O_12_	463	300, 271, 255	465	303, 229	464
Isorhamnetin-3-O-glucoside	9.46	C_21_H_20_O_11_	447	314, 285, 271, 243			448
**PHENOLIC ACIDS**							
3,4-Dihydroxybenzoic acid	3.69	C_7_H_6_O_4_	153	109,108			154
2,5-Dihydroxybenzoic acid	5.88	C_7_H_6_O_4_	153	123, 108, 95			154
6,8-di-C-glucosylapigenin	7.70	C_27_H_30_O_15_	593	503, 473, 383	595	577, 559, 511	594
4-hydroxybenzaldehyde	7.92	C_7_H_6_O_2_	121	108, 95, 93			122
12-hydroxyjasmonic acid glucoside	7.93	C_18_H_28_O_9_	387	207, 163, 119			388
Uralenneoside	4.00	C_12_H_14_O_8_	285	152, 108			286
[2-hydroxy-3-[3,4,5-trihydroxy-6-[[3,4,5-trihydroxy-6-(hydroxymethyl)oxan-2-yl]oxymethyl]oxan-2-yl]oxypropyl] hexadecanoate	14.87	C_31_H_58_O_14_	699	653, 415			654
*p*-hydroxybenzoic acid	10.00	C_7_H_6_O_3_	137	93			138
**GLYCOSYLGLYCEROLS**							
[2-hydroxy-3-[3,4,5-trihydroxy-6-[[3,4,5-trihydroxy-6-(hydroxymethyl)oxan-2-yl]oxymethyl]oxan-2-yl]oxypropyl] (9E,12E,15E)-octadeca-9,12,15-trienoate	13.90	C_33_H_56_O_14_	721	675, 415			722
**GLYCEROPHOSPHOCHOLINES**							
1-Palmitoyl-sn-glycero-3-phosphocholine	16.10	C_24_H_50_NO_7_	540	480, 255,152, 78	496	184, 125, 86	495
**LINOLEIC ACIDS AND DERIVATIVES**							
9,12,13-Trihydroxy-10,15-octadecadienoic acid	11.44	C_18_H_32_O_5_	327	291, 229			328
9,12,13-Trihydroxy-10-octadecenoic acid	11.85	C_18_H_34_O_5_	329	229, 211			330
9,10,13-Trihydroxy-10-octadecenoic acid	12.47	C_18_H_34_O_5_	329	293, 211			330
9,10-DHOME or Leukotoxin Diol	14.38	C_18_H_34_O_4_	313	277, 201			314

## Data Availability

Not applicable.

## Supplementary Materials

The following supporting information can be downloaded at: https://www.mdpi.com/article/10.3390/nu15030746/s1, Figure S1: TIC chromatograms of the *D. tortuosum* sample analyzed in negative (top) ESI mode and in positive (bottom) ESI mode.

## References

[1] Ionescu-Tucker A., Cotman C.W. (2021). Emerging roles of oxidative stress in brain aging and Alzheimer’s disease. Neurobiol. Aging..

[2] Favero G., Paganelli C., Buffoli B., Rodella L.F., Rezzani R. (2014). Endothelium and its alterations in cardiovascular diseases: Life style intervention. BioMed Res. Int..

[3] Polovina M.M., Potpara T.S. (2014). Endothelial dysfunction in metabolic and vascular disorders. Postgrad. Med..

[4] Paneni F., Beckman J.A., Creager M.A., Cosentino F. (2013). Diabetes and vascular disease: Pathophysiology, clinical consequences and medical therapy: Part I. Eur. Heart J..

[5] Ríos J.L., Francini F., Schinella G.R. (2015). Natural products for the treatment of type 2 Diabetes mellitus. Planta Med..

[6] González J., Valls N., Brito R., Rodrigo R. (2014). Essential hypertension and oxidative stress: New insights. World J. Cardiol..

[7] Song P., Zou M.H. (2014). Redox regulation of endothelial cell fate. Cell. Mol. Life Sci..

[8] Niedzielska E., Smaga I., Gawlik M., Moniczewski A., Stankowicz P., Pera J., Filip M. (2016). Oxidative stress in neurodegenerative diseases. Mol. Neurobiol..

[9] Rivas F., Poblete-Aro C., Pando M.E., Allel M.J., Fernandez V., Soto A., Nova P., Garcia-Diaz D. (2022). Effects of polyphenols in aging and neurodegeneration associated with oxidative stress. Curr. Med. Chem..

[10] Martín M.A., Goya L., Ramos S. (2017). Protective effects of tea, red wine and cocoa in diabetes. Evidence from human studies. Food Chem. Toxicol..

[11] Romacho T., Valencia I., Ramos-González M., Vallejo S., López-Esteban M., Lorenzo O., Cannata P., Romero A., San Hipólito-Luengo A., Gómez-Cerezo J.F. (2020). Visfatin/eNampt induces endothelial dysfunction in vivo: A role for Toll-Like Receptor 4 and NLRP3 inflammasome. Sci. Rep..

[12] Martin M.A., Ramos S. (2021). Impact of cocoa flavanols on human health. Food Chem. Toxicol..

[13] Kris-Etherton P.M., Keen C.L. (2002). Evidence that the antioxidant flavonoids in tea and cocoa are beneficial for cardiovascular health. Curr. Opin. Lipidol..

[14] Fisher N.D., Hughes M., Gerhard-Herman M., Hollenberg N.K. (2003). Flavanol rich cocoa induces nitric oxide-dependent vasodilation in healthy humans. J. Hypertens..

[15] Heiss C., Dejam A., Kleinbongard P., Schewe T., Sies H., Kelm M. (2003). Vascular effects of cocoa rich in flavan-3-ols. J. Am. Med. Assoc..

[16] Ciumarnean L., Milaciu M.V., Runcan O., Vesa S.C., Rachis A.L., Negrean V., Perné M.-G., Donca V.I., Alexescu T.-G., Para I. (2020). The effects of flavonoids in cardiovascular diseases. Molecules.

[17] Ebaditabar M., Djafarian K., Saeidifard N., Shab-Bidar S. (2020). Effect of dark chocolate on flow-mediated dilatation: Systematic review, meta-analysis, and dose–response analysis of randomized controlled trials. Clin. Nutr. ESPEN.

[18] Bravo L., Mateos R., Sarriá B., Baeza G., Lecumberri E., Ramos S., Goya L. (2014). Hypocholesterolaemic and antioxidant effects of yerba mate (Ilex paraguariensis) in high-cholesterol fed rats. Fitoterapia.

[19] Gutiérrez-Del-Río I., López-Ibáñez S., Magadán-Corpas P., Fernández-Calleja L., Pérez-Valero Á., Tuñón-Granda M., Miguélez E.M., Villar C.J., Lombó F. (2021). Terpenoids and polyphenols as natural antioxidant agents in food preservation. Antioxidants.

[20] Palomino O.M., Gouveia N.M., Ramos S., Martín M.A., Goya L. (2017). Protective effect of Silybum marianum on endothelial cells submitted to high glucose concentration. Planta Med..

[21] de Gouveia N.M., Ramos S., Martín M.A., Spindola F., Goya L., Palomino O.M. (2017). Vochysia rufa stem bark extract protects endothelial cells against high glucose damage. Medicines.

[22] Wang S.-L., Sarriá B., Mateos R., Goya L., Bravo L. (2019). TNF-α induced inflammation in human EA.hy926 endothelial cells is prevented by yerba mate and green coffee extracts, their main hydroxycinnamic acids, and microbial metabolites. Int. J. Food Sci. Nutr..

[23] Martins T.F., Palomino O.M., Álvarez-Cilleros D., Ramos S., Goya L. (2020). Cocoa flavanols protect human endothelial cells from oxidative stress. Plant. Food Hum. Nutr..

[24] Martín M.A., Goya L., de Pascual-Teresa S. (2020). Effect of Cocoa and Cocoa Products on Cognitive Performance in Young Adults. Nutrients.

[25] Goya L., Román R.S., de Pascual-Teresa S. (2022). Polyphenols’ effect on cerebrovascular health. Curr. Med. Chem..

[26] Strother L., Miles G.B., Holiday A.R., Cheng Y., Doherty G.H. (2021). Long-term culture of SH-SY5Y neuroblastoma cells in the absence of neurotrophins: A novel model of neuronal ageing. J. Neurosci. Methods.

[27] Carballeda Sangiao N., Chamorro S., de Pascual-Teresa S., Goya L. (2021). Aqueous extract of cocoa phenolic compounds protects differentiated neuroblastoma SH-SY5Y cells from oxidative stress. Biomolecules.

[28] Palomino O., García-Aguilar A., González A., Guillén C., Benito M., Goya L. (2021). Biological actions and molecular mechanisms of Sambucus nigra L. in neurodegeneration: A cell culture approach. Molecules.

[29] Alía M., Ramos S., Mateos R., Bravo L., Goya L. (2006). Quercetin protects human hepatoma cell line (HepG2) against oxidative stress induced by tertbutyl hydroperoxide. Toxicol. Appl. Pharmacol..

[30] Denizot F., Lang R. (1986). Rapid colorimetric assay for cell growth and survival. Modifications to the tetrazolium dye procedure giving improved sensitivity and reliability. J. Immunol. Methods.

[31] Wang H., Joseph J.A. (1999). Quantifying cellular oxidative stress by dichlorofluorescein assay using microplate reader. Free Radic. Biol. Med..

[32] Barrios-Arpi L., Arias Y., Lopez-Torres B., Ramos-Gonzalez M., Ticli G., Prosperi E., Rodríguez J.L. (2022). In vitro neurotoxicity of flumethrin pyrethroid on SH-SY5Y neuroblastoma cells: Apoptosis associated with oxidative stress. Toxics.

[33] Ramakers C., Ruijter J.M., Deprez R.H.L., Moorman A.F.M. (2003). Assumption-free analysis of quantitative real-time polymerase chain reaction (PCR) data. Neurosci. Lett..

[34] Baba S., Osakabe N., Natsume N., Muto Y., Takizawa T., Terao J. (2001). In vivo comparison of the bioavailability of catechin, epicatechin and their mixture in orally administered rats. J. Nutr..

[35] Pappas E., Schaich K.M. (2009). Phytochemicals of cranberries and cranberry products: Characterization, potential health effects and processing stability. Crit. Rev. Food Sci. Nutr..

[36] Pedersen C.B., Kyle J., Jenkinson A.M., Gardner P.T., PcPhail D.B., Duthie G.G. (2000). Effects of cranberry and blueberry juice consumption on the plasma antioxidant capacity of healthy female volunteers. Eur. J. Clin. Nutr..

[37] Palomino O., Giordani V., Chowen J.A., Fernández Alonso S., Goya L. (2022). Physiological doses of oleic and palmitic acids protect human endothelial cells from oxidative stress. Molecules.

[38] Martín M.A., Ramos S., Mateos R., Serrano A.B.G., Izquierdo-Pulido M., Bravo L., Goya L. (2008). Protection of Human HepG2 Cells against Oxidative Stress by Cocoa Phenolic Extract. J. Agric. Food Chem..

[39] Martín M.A., Ramos S., Cordero-Herrera I., Bravo L., Goya L. (2013). Cocoa phenolic extract protects pancreatic beta cell viability and function against oxidative stress. Nutrients.

[40] León-González A., Mateos R., Ramos S., Martín M.A., Sarriá B., Martín-Cordero C., López-Lázaro M., Bravo L., Goya L. (2012). Chemo-protective activity and characterization of phenolic extracts from Corema album. Food Res. Int..

[41] Baeza G., Amigo-Benavent M., Sarriá B., Goya L., Mateos R., Bravo L. (2014). Green coffee hydroxycinnamic acids but not caffeine protect human HepG2 cells against oxidative stress. Food Res. Int..

[42] Martín M.A., Ramos S., Mateos R., Marais J., Bravo L., Khoo C., Goya L. (2015). Chemical characterization and chemo-protective activity of cranberry phenolic extracts in a model cell culture. Response of the antioxidant defences and regulation of signaling pathways. Food Res. Int..

[43] Castillo G., Barrios-Arpi L., Ramos-Gonzalez M., Vidal P., Gonzales-Irribarren A., Ramos-Cevallos N., Rodríguez J.L. (2022). Neurotoxicity associated with oxidative stress and inflammasome gene expression induced by allethrin in SH-SY5Y cells. Toxicol. Ind. Health.

[44] Lee M.H., Lee J.M., Jun S.H., Lee S.H., Kim N.W., Lee J.H., Ho N.Y., Mun S.H., Kim B.K., Lim B.O. (2007). The anti-inflammatory effects of Pyrolae herba extract through the inhibition of the expression of inducible nitric oxide synthase (iNOS) and NO production. J. Ethnopharmacol..

[45] Adebayo S.A., Ondua M., Shai L.J., Lebelo S.L. (2019). Inhibition of nitric oxide production and free radical scavenging activities of four South African medicinal plants. J. Inflamm. Res..

[46] Marino G., López-Otín C. (2004). Autophagy: Molecular mechanisms, physiological functions and relevance in human pathology. Cell Mol. Life Sci. CMLS.

[47] Pelicano H., Carney D., Huang P. (2004). ROS stress in cancer cells and therapeutic implications. Drug Resist. Updat..

[48] Chen Y., McMillan-Ward E., Kong J., Israels S.J., Gibson S.B. (2008). Oxidative stress induces autophagic cell death independent of apoptosis in transformed and cancer cells. Cell Death Differ..

[49] Li J.K., Ge R., Tang L., Li Q.S. (2013). Protective effects of farrerol against hydrogen-peroxide-induced apoptosis in human endothelium-derived EA.hy926 cells. Can J. Physiol. Pharmacol..

[50] Guo S., Long M., Li X., Zhu S., Zhang M., Yang Z. (2016). Curcumin activates autophagy and attenuates oxidative damage in EA.hy926 cells via the Akt/mTOR pathway. Mol. Med. Rep..

[51] Morán-Santibañez K., Vasquez A.H., Varela-Ramirez A., Henderson V., Sweeney J., Odero-Marah V., Fenelon K., Skouta R. (2019). Larrea tridentata extract mitigates oxidative stress-induced cytotoxicity in human neuroblastoma SH-SY5Y cells. Antioxidants.

[52] Jantas D., Malarz J., Le T.N., Stojakowska A. (2021). Neuroprotective properties of kempferol derivatives from maesa membranacea against oxidative stress-induced cell damage: An association with cathepsin D inhibition and PI3K/Akt Activation. Int. J. Mol. Sci..

[53] Myhrstad M.C., Carlsen H., Nordström O., Blomhoff R., Moskaug J.O. (2002). Flavonoids increase the intracellular glutathione level by transactivation of the gamma-glutamylcysteine synthetase catalytical subunit promoter. Free Rad. Biol. Med..

[54] Moridani M.Y., Scobie H., Salehi P., O’Brien P.J. (2001). Catechin metabolism: Glutathione conjugate formation catalyzed by tyrosinase, peroxidase, and cytochrome p450. Chem. Res. Toxicol..

[55] Galati G., Lin A., Sultan A.M., O’Brien P.J. (2006). Cellular and in vivo hepatotoxicity caused by green tea phenolic acids and catechins. Free Radic. Biol. Med..

[56] Battistelli M., Malatesta M., Meschini S. (2016). Oxidative stress to promote cell death or survival. Oxid. Med. Cell. Longev..

[57] Lee H.J., Spandidos D.A., Tsatsakis A., Margina D., Izotov B.N., Yang S.H. (2019). Neuroprotective effects of Scrophularia buergeriana extract against glutamate-induced toxicity in SH-SY5Y cells. Int. J. Mol. Med..

[58] Huang W.M., Liang Y.Q., Tang L.J., Ding Y.U.E., Wang X.H. (2013). Antioxidant and anti-inflammatory effects of Astragalus polysaccharide on EA.hy926 cells. Exp. Ther. Med..

[59] Ma D., Wang Z., He Z., Wang Z., Chen Q., Qin F., Zeng M., Chen J. (2023). Pine pollen extract alleviates ethanol-induced oxidative stress and apoptosis in HepG2 cells via MAPK signaling. Food Chem. Toxicol..

[60] Chu W.L., Lim Y.W., Radhakrishnan A.K., Lim P.E. (2010). Protective effect of aqueous extract from Spirulina platensisagainst cell death induced by free radicals. BMC Complement. Altern. Med..

