# Supplementary material for: Chemical Characterization, Antioxidant Capacity and Anti-Oxidative Stress Potential of South American Fabaceae Desmodium tortuosum
Source: Nutrients. 2023 Feb 1;15(3):746. doi: 10.3390/nu15030746 (PMC9921092; doi:10.3390/nu15030746)
Supplement: Supplementary file 1 [file nutrients-15-00746-s001.zip › nutrients-2199741-supplementary.pdf]

nutrients

Chemical Characterization, Antioxidant Capacity and Anti-Oxidative Stress Potential of South American Fabaceae *Desmodium tortuosum*

José-Luis Rodríguez [1,2,*], Paola Berrios [1], Zoyla-Mirella Clavo [1], Manuel Marin-Bravo [3], Luis Inostroza-Ruiz [4], Mariella Ramos-Gonzalez [1], Miguel Quispe-Solano [5], Maria S. Fernández-Alfonso [6], Olga Palomino [6] and Luis Goya [7]

1 Faculty of Veterinary Medicine, Universidad Nacional Mayor de San Marcos, Lima 15021, Peru
2 Faculty of Veterinary, Universidad Complutense de Madrid, 28040 Madrid, Spain
3 Faculty of Biological Sciences, Universidad Nacional Mayor de San Marcos, Lima 15021, Peru
4 Faculty of Pharmacy, Universidad Nacional Mayor de San Marcos, Lima 15021, Peru
5 Faculty of Engineering in Food Industries, Universidad Nacional del Centro del Perú, Huancayo 12006, Peru
6 Faculty of Pharmacy, Universidad Complutense de Madrid, 28040 Madrid, Spain
7 Department of Metabolism and Nutrition, Spanish National Research Council (CSIC), Institute of Food Science, Technology and Nutrition (ICTAN), Jose Antonio Novais 10, 28040 Madrid, Spain
* Correspondence: josero05@ucm.es

Supplementary Materials

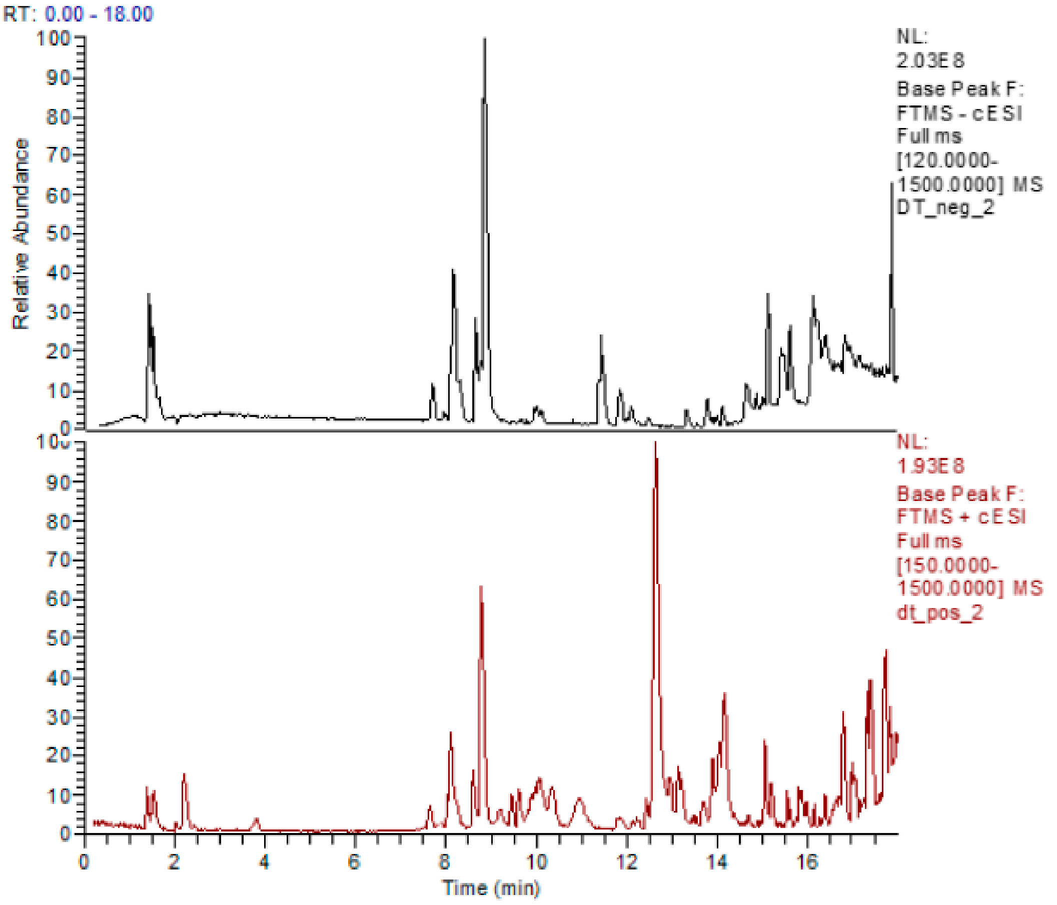

Figure S1. TIC chromatograms of the *D. tortuosum* sample analyzed in negative (top) ESI mode and in positive (bottom) ESI mode. The total ion current (TIC) chromatograms recorded for the analyzed sample are shown in Figure. The mass spectra of the compounds present in the sample were analyzed in ESI positive and ESI negative mode, and under the same chromatographic conditions. The intensity of the signals (peaks), as well as their presence or absence in the different chromatograms, varies according to the concentration and the structural characteristics of the molecules present in the sample.
